# Supplementary figures and images for: G-Protein Subunit Gamma 4 as a Potential Biomarker for Predicting the Response of Chemotherapy and Immunotherapy in Bladder Cancer
Source: Genes (Basel). 2022 Apr 14;13(4):693. doi: 10.3390/genes13040693 (PMC9027884; doi:10.3390/genes13040693)

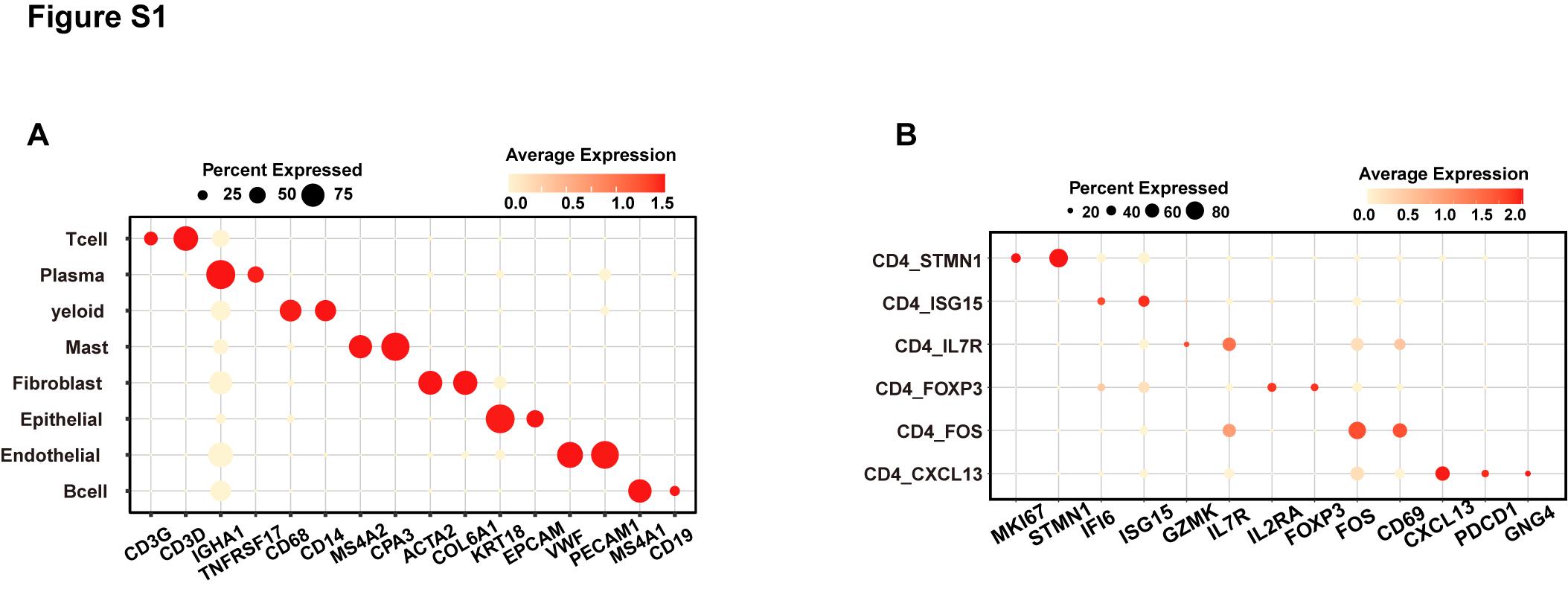

Supplement: Supplementary file 1 [file genes-13-00693-s001.zip › figure S1new.tif]

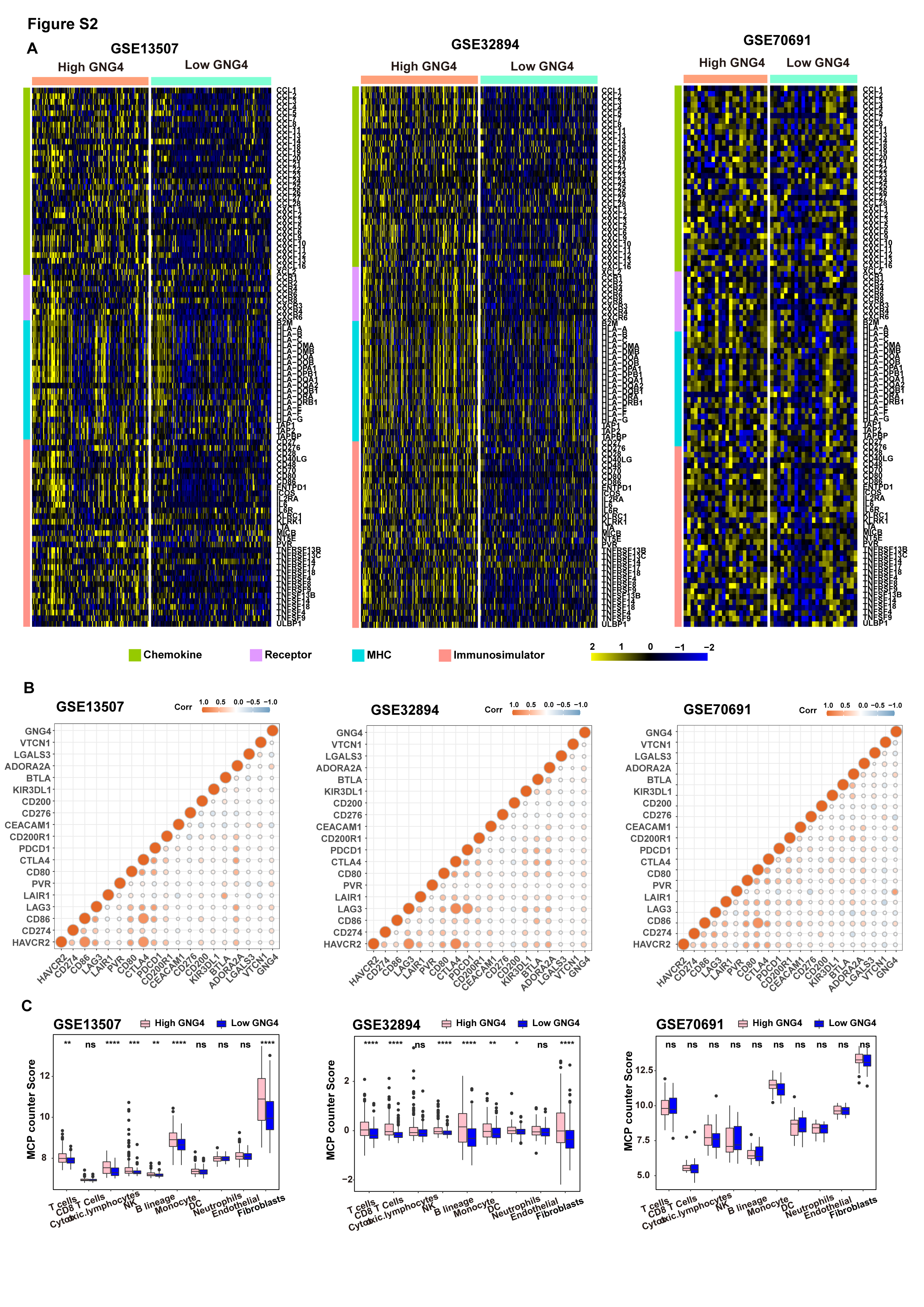

Supplement: Supplementary file 1 [file genes-13-00693-s001.zip › figure S2new.tif]

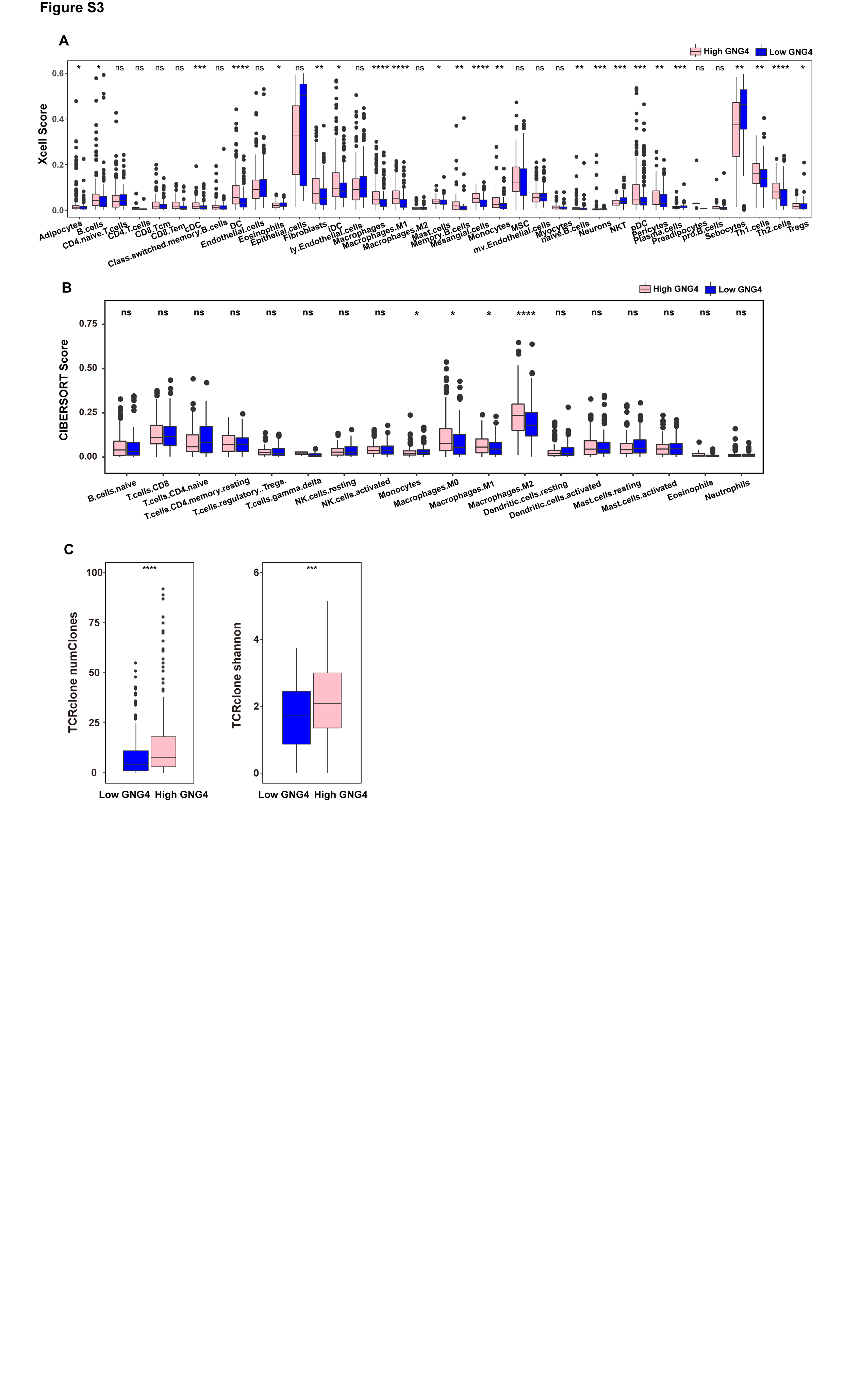

Supplement: Supplementary file 1 [file genes-13-00693-s001.zip › figure S3new.tif]

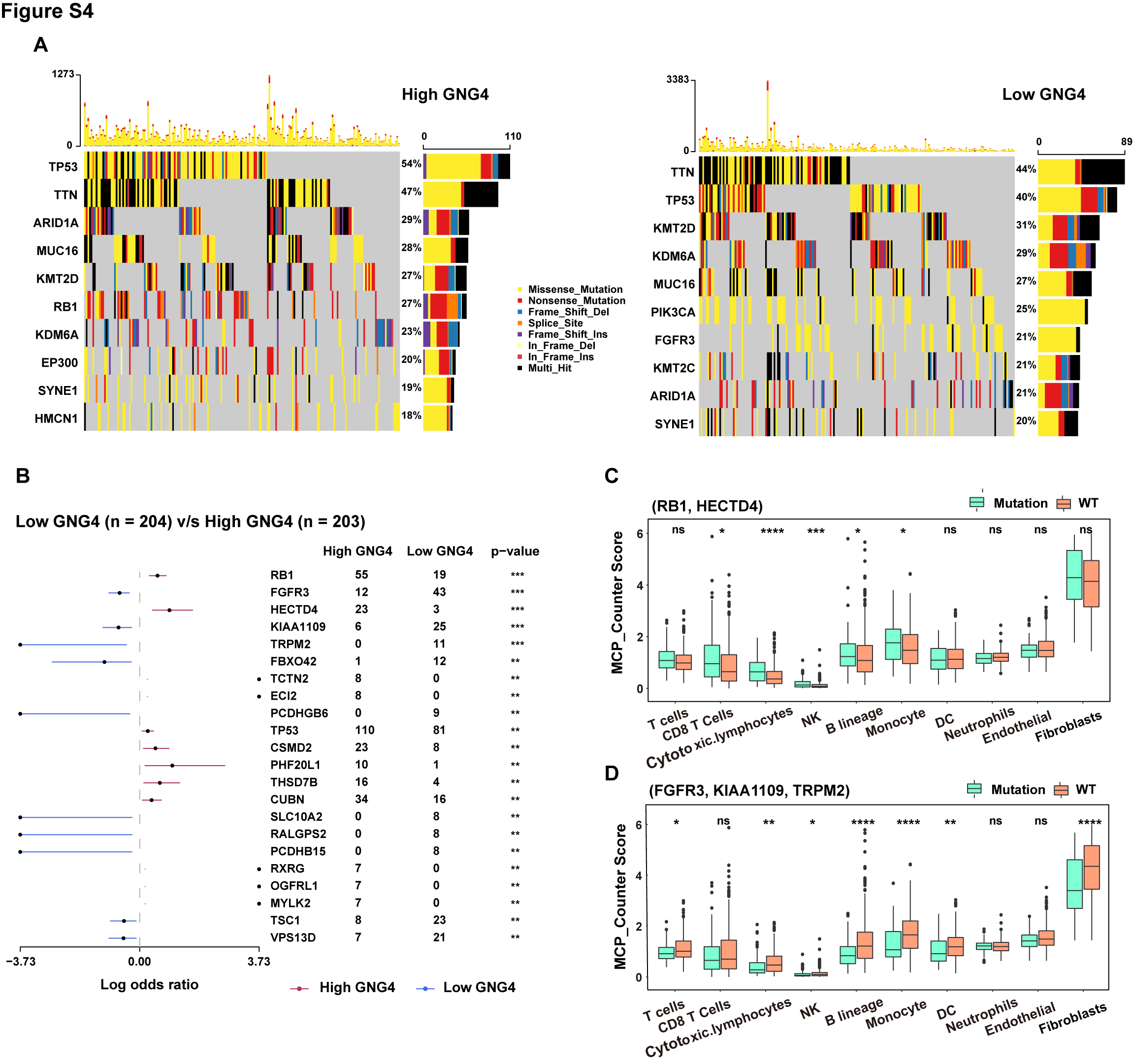

Supplement: Supplementary file 1 [file genes-13-00693-s001.zip › figure S4new.tif]
